# The Jumonji Domain-Containing Histone Demethylase Homolog 1D/lysine Demethylase 7A (JHDM1D/KDM7A) Is an Epigenetic Activator of RHOJ Transcription in Breast Cancer Cells

**DOI:** 10.3389/fcell.2021.664375

**Published:** 2021-06-23

**Authors:** Ziyu Zhang, Baoyu Chen, Yuwen Zhu, Tianyi Zhang, Xiaoling Zhang, Yibiao Yuan, Yong Xu

**Affiliations:** ^1^Department of Pathology, Jiangxi Maternal and Child Health Hospital, Nanchang, China; ^2^Key Laboratory of Targeted Invention of Cardiovascular Disease and Collaborative Innovation Center for Cardiovascular Translational Medicine, Department of Pathophysiology, Nanjing Medical University, Nanjing, China; ^3^School of Medicine, Nanchang University, Nanchang, China; ^4^Department of Gynecology, Jiangxi Provincial Maternal and Child Health Hospital, Nanchang, China; ^5^Institute of Biomedical Research, Liaocheng University, Liaocheng, China

**Keywords:** transcriptional regulation, epigenetics, histone demethylase, breast cancer cell, histone methylation

## Abstract

The small GTPase RHOJ is a key regulator of breast cancer metastasis by promoting cell migration and invasion. The prometastatic stimulus TGF-β activates RHOJ transcription *via* megakaryocytic leukemia 1 (MKL1). The underlying epigenetic mechanism is not clear. Here, we report that MKL1 deficiency led to disrupted assembly of the RNA polymerase II preinitiation complex on the RHOJ promoter in breast cancer cells. This could be partially explained by histone H3K9/H3K27 methylation status. Further analysis confirmed that the H3K9/H3K27 dual demethylase JHDM1D/KDM7A was essential for TGF-β-induced RHOJ transcription in breast cancer cells. MKL1 interacted with and recruited KDM7A to the RHOJ promoter to cooperatively activate RHOJ transcription. KDM7A knockdown attenuated migration and invasion of breast cancer cells *in vitro* and mitigated the growth and metastasis of breast cancer cells in nude mice. KDM7A expression level, either singularly or in combination with that of RHOJ, could be used to predict prognosis in breast cancer patients. Of interest, KDM7A appeared to be a direct transcriptional target of TGF-β signaling. A SMAD2/SMAD4 complex bound to the KDM7A promoter and mediated TGF-β-induced KDM7A transcription. In conclusion, our data unveil a novel epigenetic mechanism whereby TGF-β regulates the transcription of the prometastatic small GTPase RHOJ. Screening for small-molecule inhibitors of KDM7A may yield effective therapeutic solutions to treat malignant breast cancers.

## Introduction

Despite the advancement in basic and clinical research and the development of sophisticated screening techniques and interventional regimens, breast cancer, especially in malignant forms, remains one of the leading causes for cancer-related deaths in female patients in the era of personalized medicine (Shieh and Tice, 2020). It is estimated that over two million new cases of breast cancer are diagnosed, a quarter of which will eventually succumb each year worldwide (Harbeck and Gnant, 2017). Malignant breast cancers are typically characterized by uncontrolled anchor-free growth, aggressive migration/invasion, acquisition of new genetic traits, and resistance to chemotherapeutic medications. These features that distinguish malignant breast cancers from more benign ones are mirrored by profound changes in cellular transcriptome (Parsons and Francavilla, 2019; Ding et al., 2020). Whereas breast cancer cells are decidedly heterogeneous in nature, transcriptomic analyses powered by next-generation sequencing techniques have greatly facilitated the elucidation of the origins from which malignant breast cancer cells are derived and the molecular mechanisms that fuel the malignant transformation (Chaffer and Weinberg, 2010; Zhang et al., 2013; Chung et al., 2017). For instance, Chen et al. have found that, relying on single-cell RNA sequencing (scRNA-seq) data, a panel of conserved transcriptional events including those programmed by MYC and HIF-1α that help maintain stemness is likely the driving force behind malignant migration of breast cancer cells (Zhang et al., 2013). Transcriptomic analysis has also led to the discovery that metabolic reprogramming skewing the cells toward oxidative phosphorylation from glycolysis may potentially contribute to breast cancer metastasis (Davis et al., 2020).

These transcriptional events taking place in breast cancer cells, like those in any other mammalian system, are invariably influenced by the epigenetic machinery. Epigenetic regulation of transcription is mediated by differential histone and DNA modifications, ATPase-dependent chromatin remodeling, swapping of histone variants, and a host of noncoding RNAs (Goldberg et al., 2007). It is generally agreed that the chromatin status can be annotated by distinctive histone modifications: high levels of histone acetylation and H3K4 methylation typically designate transcriptional activation, whereas transcriptionally silenced regions are abounded by methylated H3K9 and methylated H3K27 (Fischle et al., 2015). Alternatively, some chromatins possess bivalent histone modifications; removal of the active modifications results in transcriptional repression, whereas erasure of the inhibitory modifications pivots to transcriptional induction (Castillo et al., 2017). By virtue of catalyzing histone demethylation, demethylases can either activate or repress transcription (Mosammaparast and Shi, 2010).

Megakaryocytic leukemia 1 (MKL1), also known as myocardin-related transcription factor A (MRTF-A), is a transcriptional regulator partnering with a host of sequence-specific transcription factors (Esnault et al., 2014). Recent investigations have pointed to an important role for MKL1 in the development and progression of breast cancer (Brandt et al., 2009), lung cancer (Cheng et al., 2015), colorectal cancer (Chen et al., 2020a), and hepatocellular cancer (Hampl et al., 2013). One of the key themes in MKL1-mediated transcriptional regulation is its ability to interact with and engage different members of the epigenetic machinery that includes histone acetyltransferases (Hanna et al., 2009; Liu et al., 2013), histone methyltransferases (Yu et al., 2014, 2017a), and histone demethylases (Lockman et al., 2007). We have previously reported that expression of RHOJ, a small GTPase belonging to the RHO family, correlates with worsened prognosis in breast cancer patients (Chen et al., 2020b). RHOJ expression is upregulated in breast cancer cells by the prometastatic stimulus TGF-β, which is mediated by MKL1. Here, we report that MKL1 interacts with the histone H3K9/27 dual demethylase JHDM1D/KDM7A to activate RHOJ transcription.

## Results

### MKL1 Deficiency Impedes the Assembly of the Preinitiation Complex by Influencing Histone Demethylation

**FIGURE 1:**
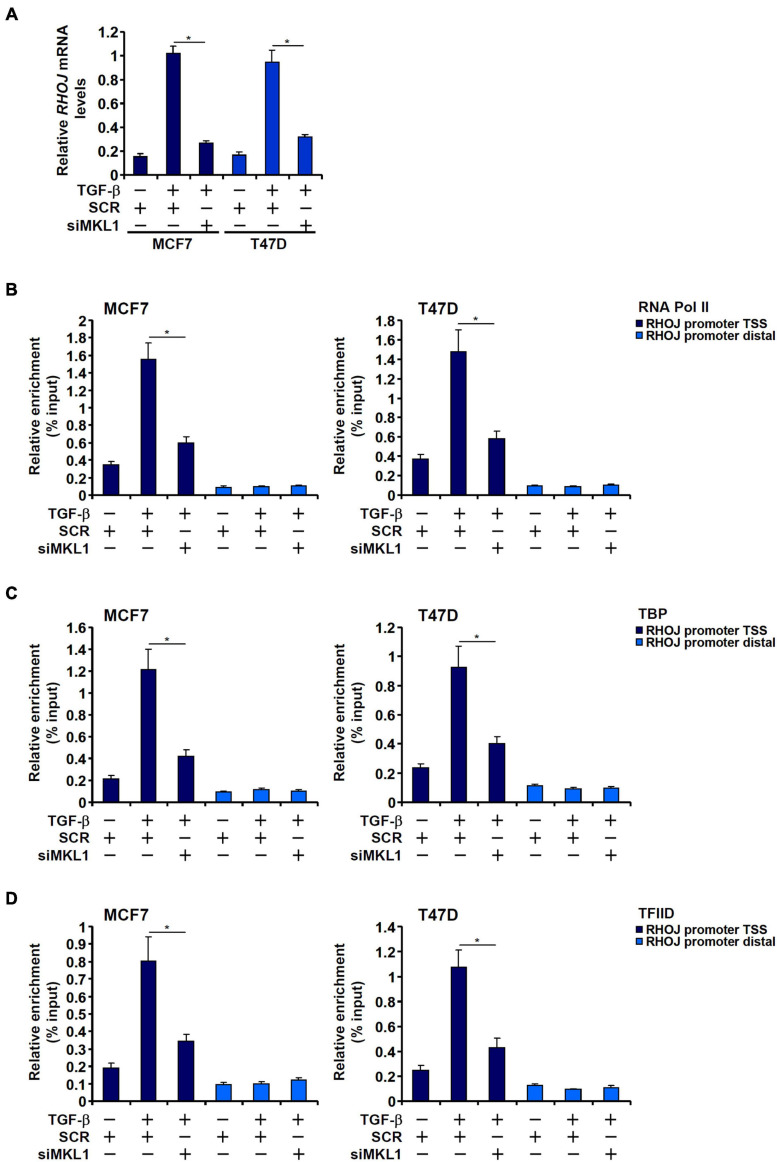
MKL1 deficiency impedes the assembly of the preinitiation complex. **(A–D)** MCF7 (left panel) and T47D (right panel) cells were transfected with indicated siRNAs followed by treatment with TGF-β for 48 h. RHOJ expression was examined by qPCR **(A)**. ChIP assays were performed with anti-RNA polymerase II **(B)**, anti-TBP **(C)**, and anti-TFIID **(D)**. All experiments were performed in triplicate wells and repeated three times, and one representative experiment is shown. Data represent mean ± SD. *p < 0.05, two-tailed *t*-test. MKL1 depletion attenuated RHOJ expression and dampened the recruitment of RNA polymerase II, TBP, and TFIID to the RHOJ promoter.

We have previously reported that MKL1 mediates RHOJ trans-activation by TGF-β in breast cancer cells (Chen et al., 2020b). We sought to determine the underlying epigenetic mechanism. Consistent with different RHOJ transcription rates (Figure 1A), TGF-β treatment prompted the assembly of the basic transcription machinery, as measured by the presence of RNA polymerase II (Figure 1B), TBP (Figure 1C), and TFIID (Figure 1D) on the RHOJ transcription start site (TSS). MKL1 knockdown, however, severely disturbed the assembly of the preinitiation complex (Figures 1B–D).

Because the PIC assembly is acutely influenced by the chromatin status, we asked whether MKL1 could impact differential histone modifications surrounding the RHOJ promoter region. We profiled the status of two well-documented histone modifications known to demarcate actively transcribed chromatin, acetylated H3 (H3Ac) and trimethylated H3K4 (H3K4Me3), and two for silenced chromatin, dimethylated H3K9 (H3K9Me2) and dimethylated H3K27 (H3K27Me2), surrounding the RHOJ promoter. We focused on the H3K9Me2 and H3K27Me2 rather than other repressive histone modifications because (1) ChIP-seq studies have provided strong correlation between dynamic changes in genome-wide H3K9Me2/H3K27Me2 levels and breast cancer metastasis (Zhao et al., 2016; Sato et al., 2017; Hou et al., 2020) and (2) the enzymes involved in H3K9me2/H3K27me2 are considered prime targets in breast cancer therapeutics (Casciello et al., 2015; Yamagishi and Uchimaru, 2017).

**FIGURE 2:**
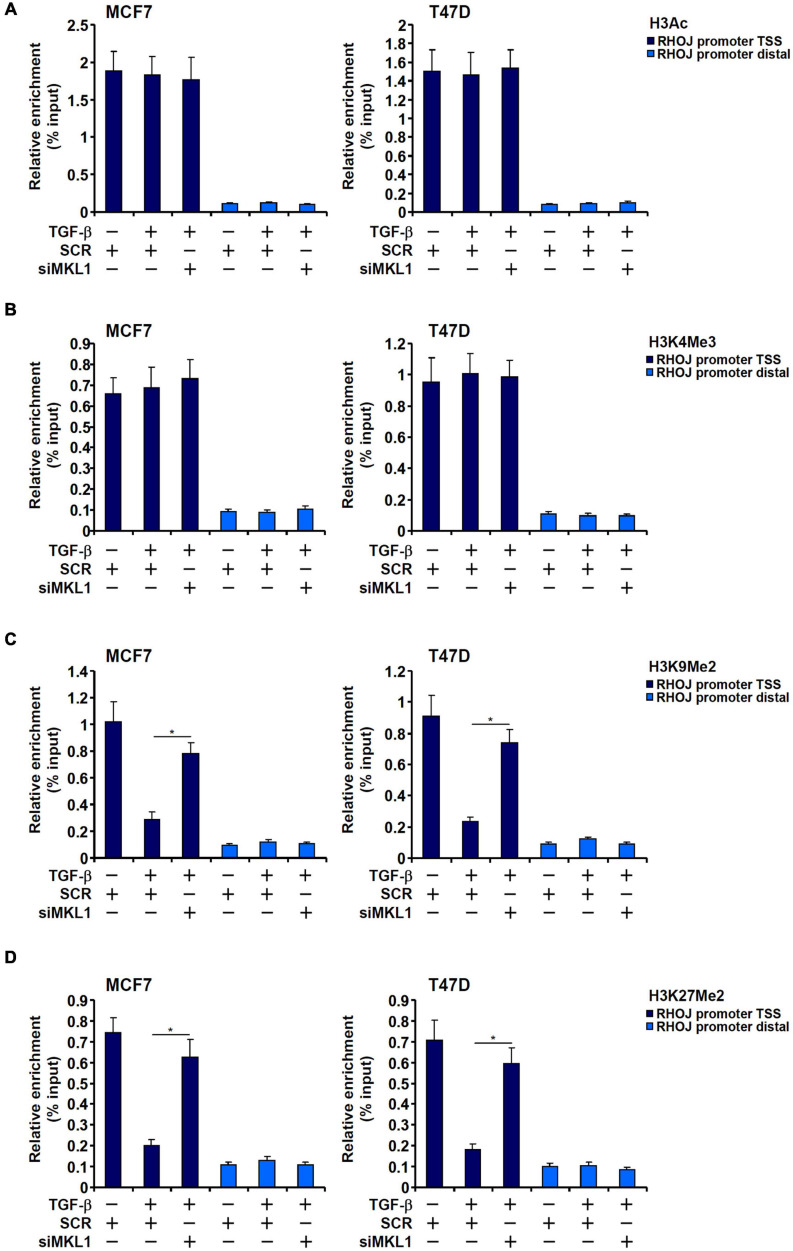
H3K9 dimethylation and H3K27 dimethylation explain differential RHOJ expression. **(A–D)** MCF7 (left panel) and T47D (left panel) cells were transfected with indicated siRNAs followed by treatment with TGF-β for 48 h. ChIP assays were performed with anti-H3Ac **(A)**, anti-H3K4Me3 **(B)**, anti-H3K9Me2 **(C)**, and anti-H3K27Me2 **(D)**. All experiments were performed in triplicate wells and repeated three times, and one representative experiment is shown. Data represent mean ± SD. *p < 0.05, two-tailed *t*-test. MKL1 depletion partially restored H3K9/H3K27 dimethylation without altering H3 acetylation or H3K4 trimethylation on the RHOJ promoter.

As shown in Figures 2A,B, TGF-β treatment marginally elevated H3Ac and H3K4Me3 levels on the RHOJ promoter, whereas MKL1 knockdown had little effect on the status of these two modifications (for MCF7 H3Ac levels, TGF-β+SCR vs. TGF-β, *p* = 0.805 and TGF-β+SCR vs. TGF-β+siMKL1, *p* = 0.801; for MCF7 H3K4Me3 levels, TGF-β+SCR vs. TGF-β, *p* = 0.722 and TGF-β+SCR vs. TGF-β+siMKL1, *p* = 0.607; for T47D H3Ac levels, TGF-β+SCR vs. TGF-β, *p* = 0.848 and TGF-β+SCR vs. TGF-β+siMKL1, *p* = 0.731; for T47D H3K4Me3 levels, TGF-β+SCR vs. TGF-β, *p* = 0.662 and TGF-β+SCR vs. TGF-β+siMKL1, *p* = 0.827). On the contrary, there was a marked decrease in H3K9Me2 levels and H3K27Me2 levels surrounding the RHOJ promoter upon TGF-β stimulation, whereas MKL1 knockdown largely blocked the removal of methylated H3K9 (Figure 2C) and H3K27 (Figure 2D). Taken together, these data suggest that MKL1 likely contributes to RHOJ trans-activation by influencing histone H3K9/K27 demethylation on the RHOJ promoter.

### JHDM1D/KDM7A Mediates TGF-β-Induced RHOJ Transcription

**FIGURE 3:**
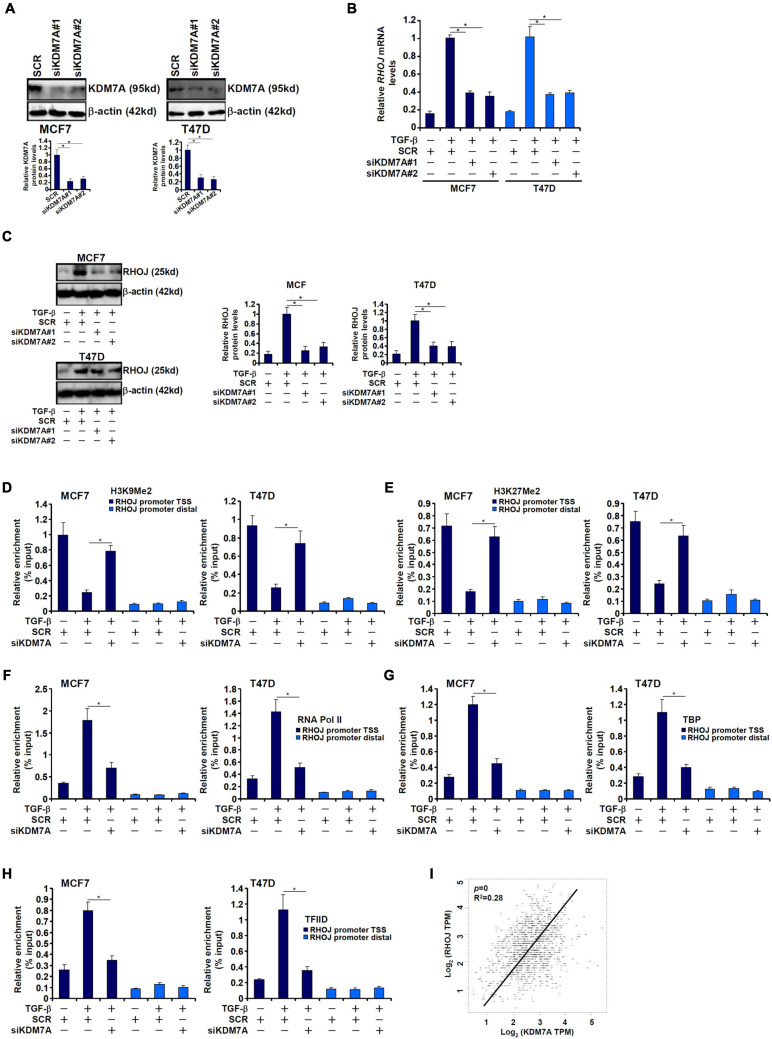
JHDM1D/KDM7A mediates TGF-β-induced RHOJ transcription. **(A–H)** MCF7 (left panel) and T47D cells (left panel) were transfected with indicated siRNAs followed by treatment with TGF-β for 48 h. Knockdown efficiencies were validated by Western blotting **(A)**. RHOJ expression was examined by qPCR **(B)** and Western blotting **(C)**. ChIP assays were performed with anti-H3K9Me2 **(D)**, anti-H3K27Me2 **(E)**, anti-RNA polymerase II **(F)**, anti-TBP **(G)**, and anti-TFIID **(H)**. All experiments were performed in triplicate wells and repeated three times, and one representative experiment is shown. Data represent mean ± SD. *p < 0.05, two-tailed *t*-test. KDM7A mediated RHOJ trans-activation by removing H3K9/H3K27 methylation to facilitate the recruitment of RNA polymerase II, TBP, and TFIID to the RHOJ promoter. **(I)** Expression data were extracted from the public database to draw the scatter plot. Pearson correlation coefficient was calculated. Positive correlation between KDM7A expression and RHOJ expression was identified in human breast cancer tissues.

The Jumonji domain-containing histone demethylase 1D, also known as lysine demethylase 7A, possesses dual specificities toward dimethylated H3K9 and dimethylated H3K27 (Huang et al., 2010). We proposed that JHDM1D/KDM7A could participate in RHOJ induction by TGF-β in breast cancer cells. Endogenous KDM7A was depleted with two separate pairs of siRNAs (Figure 3A). KDM7A knockdown markedly weakened RHOJ induction by TGF-β at mRNA (Figure 3B) and protein (Figure 3C) levels. Consistently, KDM7A silencing reversed the disappearance of H3K9Me2 (Figure 3D) and H3K27Me2 (Figure 3E) from the RHOJ promoter suggesting that KDM7A likely contributes to RHOJ trans-activation by erasing the repressive histone modifications. Of interest, KDM7A knockdown partially blocked the accessibility of the RHOJ promoter to the basic transcriptional machinery including RNA polymerase II (Figure 3F), TBP (Figure 3G), and TFIID (Figure 3H), suggesting that KDM7A may modulate the chromatin structure to influence the PIC assembly. In addition, expression data extracted from the public cancer database (TCGA) showed a highly correlative relationship between KDM7A expression and RHOJ expression in human breast cancer tissues (Figure 3I).

### MKL1 Recruits JHDM1D/KDM7A to the RHOJ Promoter

**FIGURE 4:**
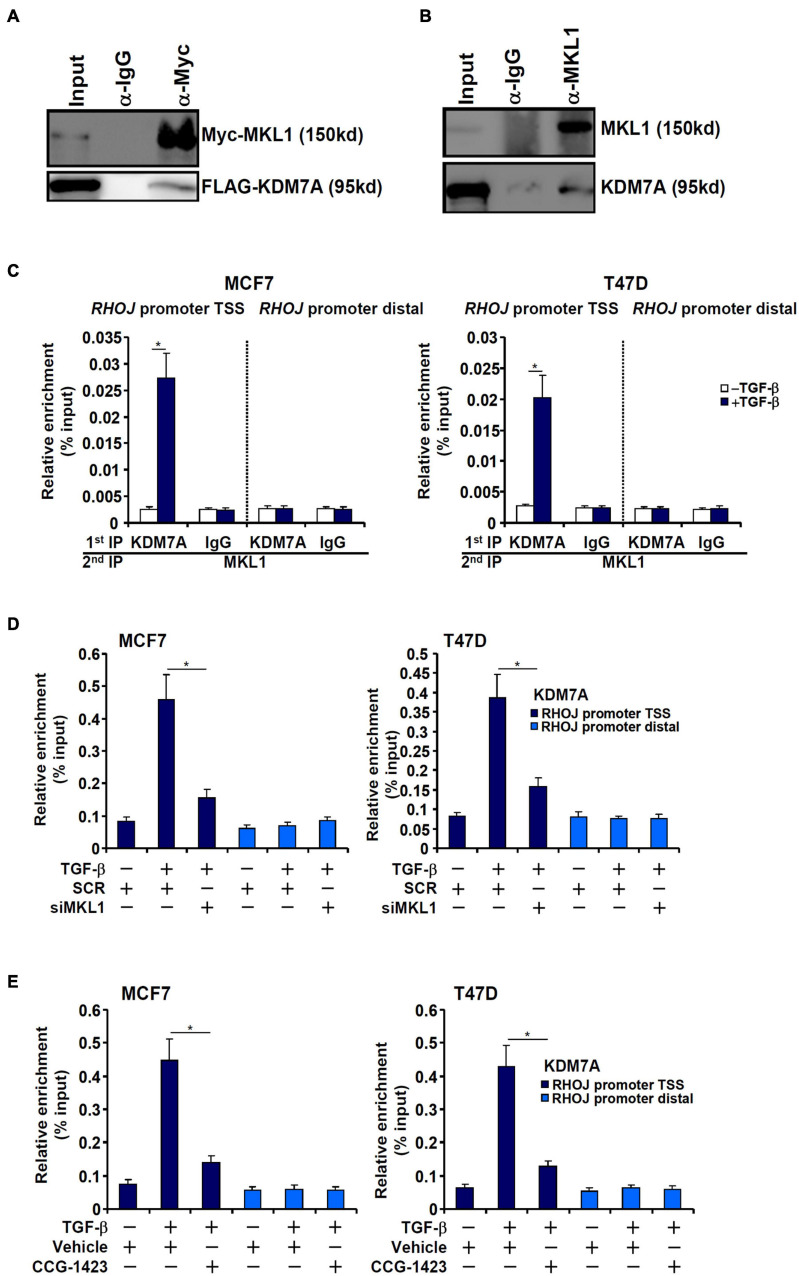
MKL1 recruits JHDM1D/KDM7A to the RHOJ promoter. **(A)** FLAG-tagged KDM7A and Myc-tagged MKL1 were transfected into HEK293 cells. Immunoprecipitation was performed with anti-Myc or IgG. **(B)** MCF7 whole-cell lysates were immunoprecipitated with anti-MKL1 or IgG. **(C)** MCF7 (left panel) and T47D cells (left panel) were treated with or without TGF-β for 48 h. Re-ChIP was performed with indicated antibodies. **(D)** MCF7 (left panel) and T47D cells (left panel) were transfected with indicated siRNAs followed by treatment with TGF-β for 48 h. ChIP assay was performed with anti-KDM7A. **(E)** MCF7 (left panel) and T47D cells (left panel) were treated with TGF-β in the presence or absence of CCG-1423 (10 μM) for 48 h. ChIP assay was performed with anti-KDM7A. All experiments were performed in triplicate wells and repeated three times, and one representative experiment is shown. Data represent mean ± SD. *p < 0.05, two-tailed *t*-test. MKL1 interacted with KDM7A and depletion/inhibition of MKL1 reduced KDM7A recruitment to the RHOJ promoter.

Both MKL1 and KDM7A seemed to be essential for RHOJ trans-activation in breast cancer cells raising the possibility that MKL1 might interact with and recruit KDM7A to the RHOJ promoter. We performed the following experiments to verify this hypothesis. Co-immunoprecipitation assays showed that ectopically expressed MKL1/KDM7A in HEK293 cells (Figure 4A) and endogenous MKL1/KDM7A in MCF7 cells (Figure 4B) interacted with each other. Re-ChIP assay confirmed that TGF-β treatment promoted the interaction between MKL1 and KDM7A (Figure 4C). In addition, MKL1 knockdown by siRNA (Figure 4D) or MKL1 inhibition by a small-molecule compound CCG-1423 (Evelyn et al., 2007) (Figure 4E) dampened the association of KDM7A with the RHOJ promoter.

### KDM7A Promotes Breast Cell Migration and Invasion *in vitro* and *in vivo*

**FIGURE 5:**
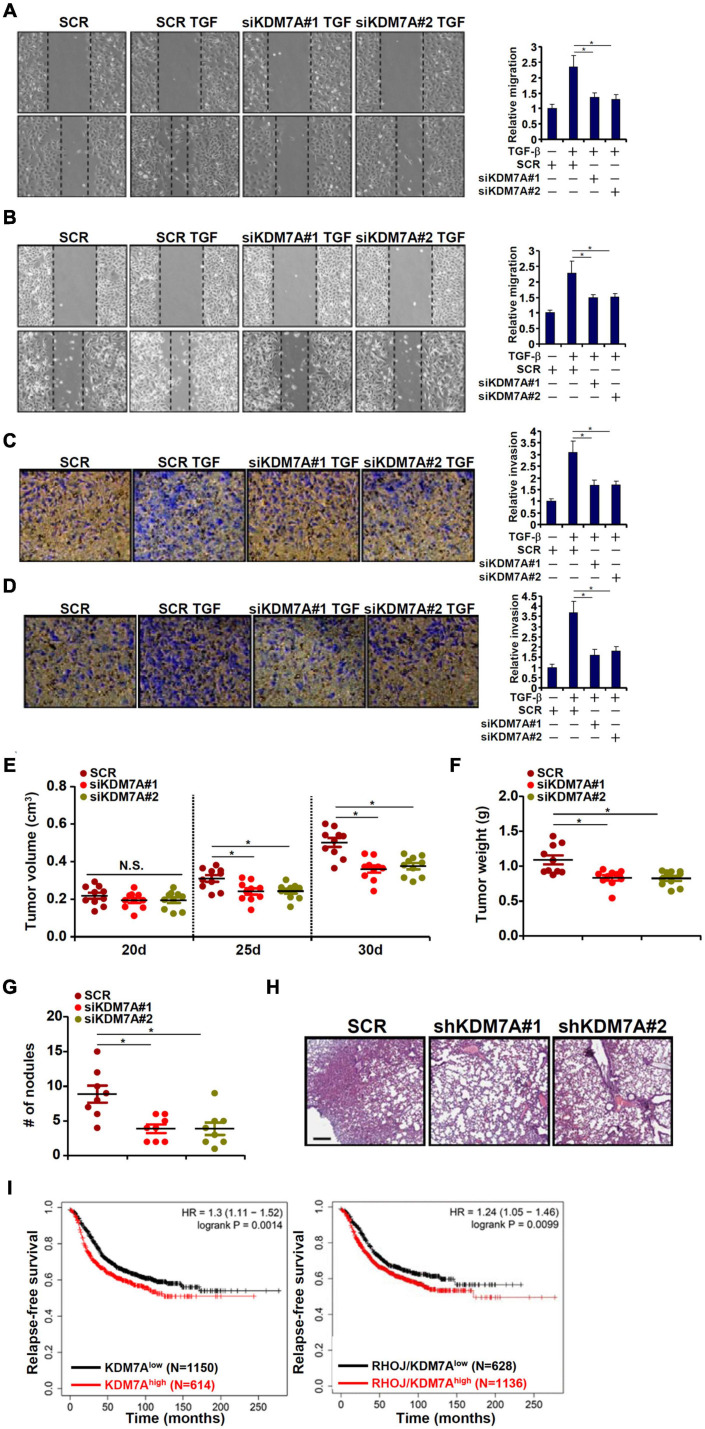
KDM7A promotes breast cell migration and invasion *in vitro* and *in vivo*. **(A,B)** MCF7 cells **(A)** and T47D cells **(B)** were transfected with indicated siRNAs followed by TGF-β treatment for 48 h. When the cells reached confluence, scratch wound was created by using a sterile micropipette tip. Photos were taken at 0 and 24 h after wound creation. The changes in side-to-side wound area were measured by Image-Pro. Data were expressed as % migration compared with control arbitrarily set as 100%. **(C,D)** MCF7 cells **(C)** and T47D cells **(D)** were transfected with indicated siRNAs. Cell invasion was examined by transwell assay. All experiments were performed in triplicate wells and repeated three times, and one representative experiment is shown. **(E,F)** Heterotopic xenograft assay was performed as described in the section “Materials and Methods.” *N* = 10 mice for each group. **(G,H)** *In vivo* metastasis was performed as described in the section “Materials and Methods.” *N* = 8 mice for each group. Data represent mean ± SD. *p < 0.05, two-tailed *t*-test. KDM7A depletion attenuated migration/invasion of breast cancer cells *in vitro* and partially blockaded metastasis of breast cancer cells *in vivo*. **(I)** Kaplan–Meier plot of survival in breast cancer patients with high and low KDM7A expression either singularly or in combination of RHOJ expression. Breast cancer patients with higher KDM7A/RHOJ expression displayed poorer survival.

We next evaluated the functional relevance of KDM7A in the migration/invasion of breast cancer cells *in vitro* and *in vivo*. Wound healing assay (Figures 5A,B) and transwell assay (Figures 5C,D) demonstrated that KDM7A knockdown blockaded TGF-β-induced breast cancer cell migration and invasion. Next, two different animal models were exploited to evaluate the effect of KDM7A knockdown on breast cancer cell migration/invasion *in vivo*. In the first model, stable MCF7 cells were inoculated subcutaneously into the nude mice. Although the amplification of tumor volume was not altered by KDM7A knockdown early on following the inoculation, it was significantly slowed toward the end (Figure 5E). Consistently, when the mice were sacrificed, it was discovered that tumor weight was significantly smaller in mice receiving the inoculation of KDM7A-depleted cells than the control cells (Figure 5F). It should be pointed out that the observed phenotype could be accounted for by, in addition to altered invasiveness/migration, skewed breast cancer cell proliferation and/or survival. In the second model, the cells were injected into the tail veins and the mice were sacrificed 5 weeks later to evaluate the formation of tumorous nodules in the lungs. KDM7A silencing significantly suppressed the metastatic abilities of breast cancer cells (Figures 5G,H). More importantly, patient data extracted from the TCGA database indicated that high KDM7A expression, either singularly or in combination of high RHOJ expression, predicted poor prognosis (Figure 5I).

### JHDM1D/KDM7A Is Transcriptionally Activated by TGF-β in Breast Cancer Cells

**FIGURE 6:**
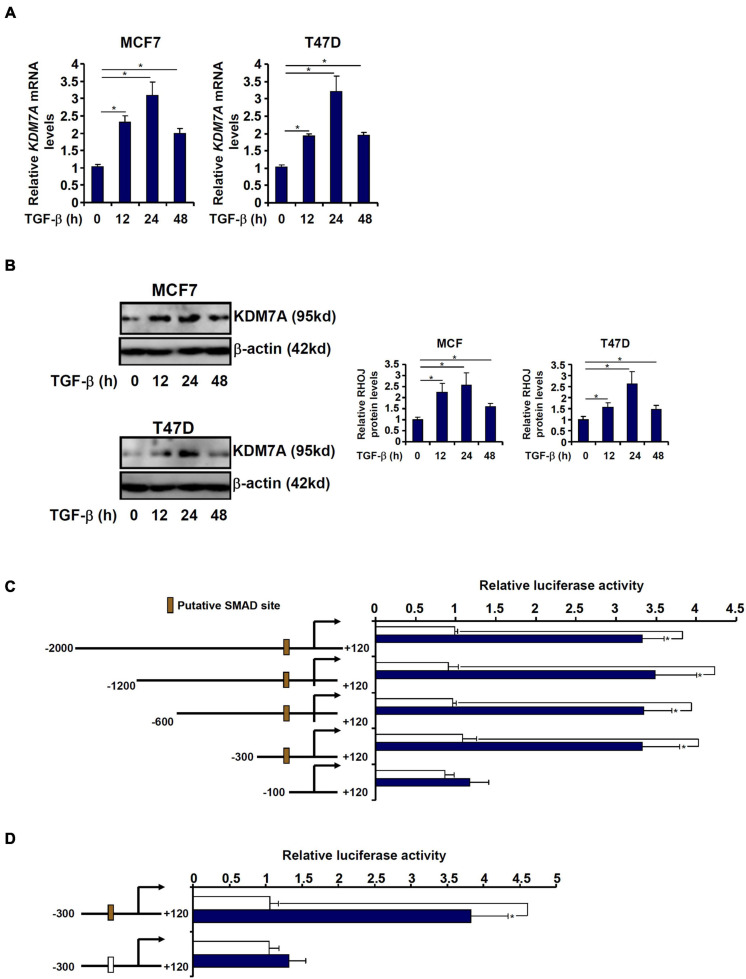
JHDM1D/KDM7A is transcriptionally activated by TGF-β in breast cancer cells. **(A,B)** MCF7 (left panel) and T47D cells (left panel) were treated with TGF-β and harvested at indicated time points. KDM7A expression was examined by qPCR and Western blotting. **(C)** Human KDM7A promoter constructs were transfected into MCF7 cells followed by treatment with TGF-β. Luciferase activities were normalized by protein concentration and GFP fluorescence. Data are expressed relative luciferase activities compared with the control group. **(D)** Wild-type and mutant human KDM7A promoter constructs were transfected into MCF7 cells followed by treatment with TGF-β. Luciferase activities were normalized by protein concentration and GFP fluorescence. Data are expressed as relative luciferase activities compared with the control group. All experiments were performed in triplicate wells and repeated three times, and one representative experiment is shown. Data represent mean ± SD. *p < 0.05, two-tailed *t*-test.

Exposure of breast cancer cells to TGF-β led to an upregulation of KDM7A expression at both mRNA (Figure 6A) and protein (Figure 6B) levels. We asked whether the activation of KDM7A by TGF-β occurred at the transcriptional level. Genomic DNA spanning approximately 2 kb of the human *KDM7A* promoter (-2,000/+120) was cloned and fused to a luciferase reporter (Figure 6C). When the *KDM7A* promoter-luciferase was transfected into MCF7 cells followed by treatment with TGF-β, it was discovered that TGF-β augmented the promoter activity by more than 3× fold (Figure 6C), suggesting that TGF-β could indeed directly regulate KDM7A transcription. In order to delineate the TGF-response element within the *KDM7A* promoter, serial inward deletions were introduced to the full-length construct to create shorter constructs. The deletions did not impact the activation of the *KDM7A* promoter by TGF-β until and unless ∼200 bp (-300/-100) from the most proximal *KDM7A* promoter were removed (Figure 6C). A closer look at this region revealed a putative GC-rich SMAD binding site (-189/-184) that could potentially mediate the TGF response. Mutation of this region completely abrogated TGF-β-induced activation of the *KDM7A* promoter (Figure 6D).

### The SMAD2/SAMD4 Complex Mediates Transcriptional Activation of JHDM1D/KDM7A in Breast Cancer Cells

**FIGURE 7:**
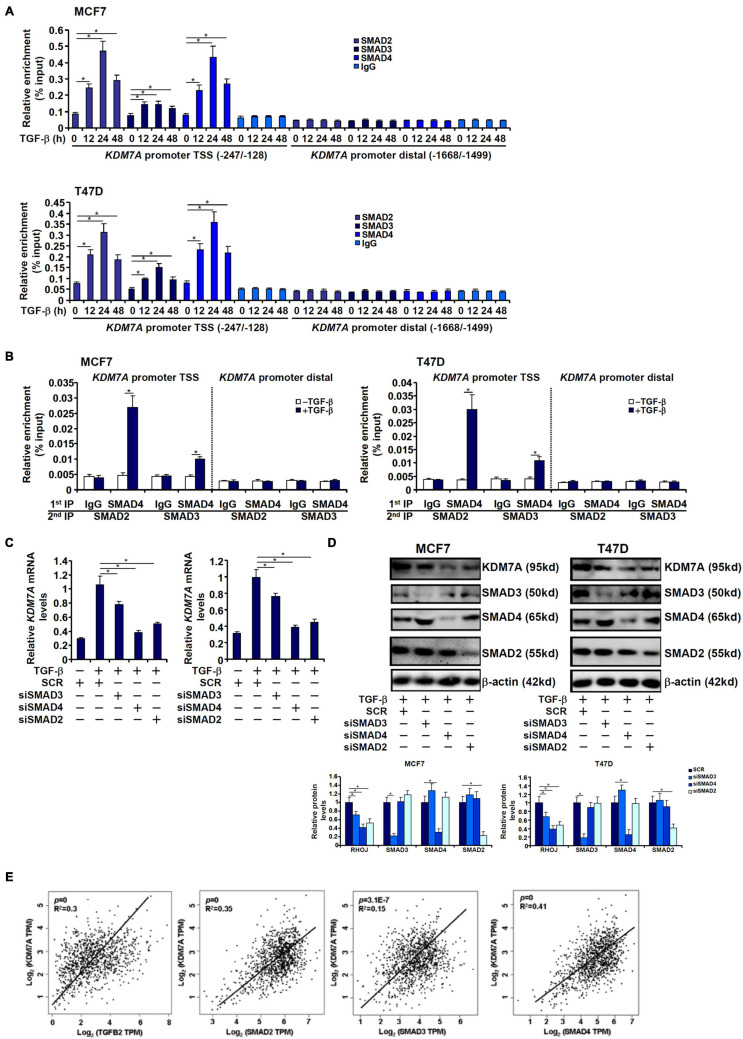
SMAD2/SAMD4 complex mediates transcriptional activation of JHDM1D/KDM7A in breast cancer cells. **(A)** MCF7 (left panel) and T47D cells (left panel) were treated with TGF-β and harvested at indicated time points. KDM7A expression was examined by qPCR and Western blotting. ChIP assay was performed with anti-SMAD2, anti-SMAD3, anti-SMAD4, or IgG. **(B)** MCF7 (left panel) and T47D cells (left panel) were treated with TGF-β and harvested at indicated time points. KDM7A expression was examined by qPCR and Western blotting. Re-ChIP assay was performed with indicated antibodies. **(C,D)** MCF7 (left panel) and T47D cells (left panel) were transfected with siRNAs targeting SMAD or scrambled siRNA followed by treatment with TGF-β. KDM7A expression was examined by qPCR and Western blotting. All experiments were performed in triplicate wells and repeated three times, and one representative experiment is shown. Data represent mean ± SD. *p < 0.05, two-tailed *t*-test. **(E)** Expression data were extracted from the public database to draw the scatter plot. Pearson correlation coefficient was calculated. Positive correlation between KDM7A expression and SMAD2/3/4 expression was identified in human breast cancer tissues.

Because receptor-associated SMADs (SMAD2/SMAD3) typically form a heterodimeric complex with the common SMAD (SMAD4) to mediate the transcription of TGF target genes, we next analyzed the transcriptional mechanism whereby SMAD proteins contribute to KDM7A trans-activation. ChIP assay performed in both MCF7 cells and T47D cells showed that TGF-β treatment stimulated the recruitment of SMAD proteins to the *KDM7A* proximal promoter with distinctive patterns: SMAD2 and SMAD4 occupied the *KDM7A* promoter much more strongly than SMAD3 peaking at 24 h after TGF-β treatment (Figure 7A). Furthermore, Re-ChIP assay confirmed that a SMAD2/SMAD4 complex was clearly detectable on the *KDM7A* promoter, whereas the interaction between SMAD3 and SMAD4 on the *KDM7A* promoter was much weaker by comparison (Figure 7B). Consistently, knockdown of SMAD2 or SMAD4 resulted in substantial decrease of KDM7A expression, whereas SMAD3 knockdown had a more moderate effect (Figures 7C,D). Finally, by comparing the expression patterns of components of the TGF signaling pathway and KDM7A in human breast cancer specimens, we discovered that there was statistically significant correlation between KDM7A and TGFB2/SMAD2/SMAD3/SMAD4 (Figure 7E).

## Discussion

The ascendancy of the epigenomic era has not only greatly broadened the understanding of cancer pathogenesis but enabled the identification of druggable targets to treat malignant cancers (Brower, 2011; Dawson and Kouzarides, 2012). In the present study, we delineate a novel pathway wherein MKL1 recruits the histone H3K9/H3K27 demethylase JHDM1D/KDM7A to regulate breast cancer metastasis through epigenetically activating RHOJ transcription. MKL1 has a well-documented reputation of being the link between epigenetic machinery and the sequence-specific transcription factors. For instance, MKL1, *via* recruiting the H3K9 demethylase JMJD1A and the chromatin remodeling protein BRG1/BRM, activates SRF-dependent transcription of smooth muscle cell differentiation marker genes to maintain the contractile phenotype of SMCs (Lockman et al., 2007; Zhang et al., 2007). The cultivation of a proinflammatory phenotype in endothelial cells (Fang et al., 2011; Yang et al., 2013; Weng et al., 2015a,b), smooth muscle cells (Yang et al., 2014), and macrophages (Yu et al., 2014, 2017a) by MKL1 appears to be mediated by its interaction with the H3K4 methyltransferase complex COMPASS, which functions to fine-tune the affinity of NF-κB for target promoters. MKL1 is also able to enlist acetyltransferases, including CBP (Hanna et al., 2009), p300 (Xu et al., 2015), and MYST1 (Liu et al., 2018; Yu et al., 2018), to regulate a wide range of pathophysiological processes. Thus, though our analysis was confined to a single locus (RHOJ), the data and implications are consistent with the transcription-modulatory role MKL1 plays in the pathogenesis of human diseases and, therefore, may well be extrapolated to a broader context. A caveat, however, needs to be pointed out regarding the current model. Because histone and non-histone factors share the same set of modifying enzymes, the possibilities that some of the MKL1-interacting epigenetic enzymes may directly use MKL1 as substrate to modulate its activity and target gene transcription remains unexplored. Indeed, we have previously shown that MKL1 acetylation levels are influenced by the acetyltransferase PCAF (Yu et al., 2017b) and the deacetylase HDAC5 (Li et al., 2017). Few non-histone substrates have been identified for KDM7A and even less is understood regarding the functional relevance of these modifications. Future investigations should continue to solve this lingering issue.

Here, we show that a locus-specific histone H3K9/H3K27 demethylase activity, presumably conferred to by KDM7A, is synonymous with the accessibility of the basal transcriptional machinery. It is generally believed that H3K4 methylation on gene promoters, but not H3K9/H3K27 methylation, is compatible with RNA Pol II occupancy and transcriptional initiation. Previously, it has been demonstrated that transcription activation of target promoters by MKL1 is associated with an H3K4 methyltransferase activity (Weng et al., 2015b), which presumably leads to the assembly and stabilization of the basal transcriptional machinery. Of note, unlike previously identified target promoters for MKL1 (e.g., proinflammatory mediators), recruitment of an H3K4 methyltransferase activity or an acetyltransferase activity did not appear to be the rate-limiting step for RHOJ trans-activation; instead, MKL1 relied on the H3K9/H3K27 demethylase activity, provided by KDM7A, to alter the chromatin structure for the basal transcriptional machinery. It remains undetermined how MKL1 chooses between different histone-modifying enzymes in a locus-specific manner to regulate transcription. Of interest, genome-wide binding patterns of MKL1 detected in fibroblast cells (Esnault et al., 2014) and macrophages (Xie, 2014) using ChIP-seq (the latter examined the binding of an ectopically expressed MKL1 with a tag antibody) showed that MKL1 activation is coincident with RNA Pol II recruitment and promoter clearance. It is unknown, however, whether the MKL1 target promoters are stratified by the differential chromatin microenvironment. We have previously shown, with ChIP-seq, that MKL1-dependent H3K4 trimethylation selectively marks the promoters from which proinflammatory mediators are transcribed (Yu et al., 2017a). The idea that active H3K9/H3K27 demethylation may be associated with a subgroup of functionally related (e.g., promigration/invasion) MKL1 target promoters during cancer metastasis is tempting and deserves further investigation.

An interesting finding is that TGF-β stimulates RHOJ transcription not only by promoting the recruitment of KDM7A but its availability. Of note, this is not the first instance where TGF-β appears to epigenetically regulate transcription by controlling the overall pool of histone-modifying enzymes. Liu et al. have reported that TGF-β-induced epithelial–mesenchymal transition is paralleled and potentially mediated by a group of histone demethylases including KDM5B, KDM6B, and KDM7A (Liu et al., 2019). TGF-β-induced chondrogenic differentiation of mesenchymal stem cells is achieved, at least in part, through upregulation of KDM4B expression (Lee et al., 2016). In gastric cancer cells, TGF-β, *via* SMAD3, stimulates the expression of the histone demethylase RBP2, which reciprocally augments SMAD3 activity (Liang et al., 2015). These observations point to a common theme of TGF-β-induced prometastatic transcriptional programs in which histone-modifying enzymes function as both a downstream target and a central mediator. It should be pointed out that despite the statistically significant correlation between KDM7A expression and SMAD expression being recorded, the correlation was relatively weak (*R*^2^ = 0.15–0.4), which indicates that alternative mechanisms, in addition to the one proposed here, might contribute to the regulation of KDM7A transcription in breast cancer cells and need to be addressed in future studies.

Although we show here that KDM7A is potentially important for the malignant spread of breast cancer cells, this observation is unlikely to be easily explained by its activation of RHOJ transcription. In particular, although statistically significant correlation between KDM7A expression and RHOJ expression was observed, the correlation was not very strong (*R*^2^ = 0.28). In addition, the survival advantage of combined low KDM7A/RHOJ expression was less significant than that of low KDM7A alone (Figure 5I), suggesting that factors other than KDM7A might play a more dominant role governing RHOJ trans-activation in malignant breast cancers. These observations also suggest that KDM7A and RHOJ may possess mutually independent prometastatic functions. Prior studies, based on either single-locus or genome-wide gene expression analysis, have suggested that KDM7A is involved in the regulation of target genes downstream of the NF-κB pathway (Higashijima et al., 2020) and the Wnt/β-catenin pathway (Yang et al., 2019a; Liu et al., 2020), which allude to a broader promalignancy mechanism. Small-molecule inhibitors of KDM7A have been discovered although their therapeutic potentials in breast cancer have yet to be explored (Gerken et al., 2017). Our report as summarized here undoubtedly provides a strong rationale for considering these compounds as a viable treatment option for the most malignant forms of breast cancer.

## Materials and Methods

### Cell Culture

Human breast cancer cells (MCF7 and T47D) were obtained from and authenticated by the Chinese Academy of Sciences Type Culture Collection Cell Bank and were maintained in DMEM (Invitrogen) as previously described (Chen et al., 2020b). Human recombinant TGF-β was purchased from R&D. CCG-1423 was purchased from Selleck. Stable cells were generated as previously described (Chen et al., 2020a). FLAG-tagged KDM7A (Lee et al., 2018) and Myc-tagged MKL1 (Wu T. et al., 2020) have been previously described. Small interfering RNA sequences were purchased from Dharmacon: for human MKL1, GUGUCUUGGUGUAGUGU; for human KDM7A#1, UGAACAUGCCUUUGAAAUUUU; for human KDM7A#2: CTTTGAGGCTTCAAGAGAGCCTCAAAG; for human SMAD2, GUCCCAUGAAAAGACUUAA; for human SMAD3, GGAGAAAUGGUGCGAGAAG; and for human SMAD4, GUACUUCAUACCAUGCCGA. Transient transfections were performed with Lipofectamine 2000 (Invitrogen). Luciferase activities were assayed 24–48 h after transfection using a luciferase reporter assay system (Promega) as previously described (Chen et al., 2020a,c; Li et al., 2020a).

### RNA Isolation and Real-Time PCR

RNA was extracted with the RNeasy RNA isolation kit (Qiagen) as previously described (Lv et al., 2020; Mao et al., 2020; Yang et al., 2020a,b). Reverse transcriptase reactions were performed using a SuperScript First-strand Synthesis System (Invitrogen). Real-time PCR reactions were performed on an ABI Prism 7500 system with the following primers: human *RHOJ*, 5′-CGGCTGCAATGGACATGAG-3′ and 5′-GGCACGTATTCCTCTGGGAAG-3′; human KDM7A, 5′-GCGGCTCAAGCCTTCAGAAT-3′ and 5′-TGCCTGGTTGCTGATAGGTG-3′. Ct values of target genes were normalized to the Ct values of a housekeeping control gene (18s, 5′-CGCGGTTCTATTTTGTTGGT-3′ and 5′-TCGTCTTCGAAACTCCGACT-3′) using the ΔΔCt method and expressed as relative mRNA expression levels compared with the control group which is arbitrarily set as 1. All experiments were performed in triplicate wells and repeated three times.

### Protein Extraction, Immunoprecipitation, and Western Blotting

Whole-cell lysates were obtained by resuspending cell pellets in RIPA buffer (50 mM Tris pH 7.4, 150 mM NaCl, 1% Triton X-100) with freshly added protease inhibitor (Roche) as previously described (Dong et al., 2020; Fan et al., 2020; Li et al., 2020b,c). Specific antibodies or preimmune IgGs (P.I.I.) were added to and incubated with cell lysates overnight before being absorbed by Protein A/G-plus Agarose beads (Santa Cruz). Precipitated immune complex was released by boiling with 1× SDS electrophoresis sample buffer. Western blot analyses were performed with anti-MKL1 (Proteintech, 21166-1), anti-KDM7A (Genetex, GTX32688), anti-SMAD2 (Proteintech, 12570-1), anti-SMAD3 (Abcam, ab208182), anti-SMAD4 (Proteintech, 10231-1), anti-RHOJ (Sigma, HPA003050), and anti-β-actin (Sigma, A2228) antibodies. All experiments were repeated three times.

### Chromatin Immunoprecipitation and Re-ChIP

Chromatin immunoprecipitation (ChIP) assays were performed essentially as described before (Li et al., 2019a,b,c,d,e; Lu et al., 2019; Shao et al., 2019; Weng et al., 2019; Sun et al., 2020; Wu X. et al., 2020; Dong et al., 2021; Hong et al., 2021; Kong et al., 2021). Briefly, chromatin was cross-linked with 1% formaldehyde. Cells were incubated in lysis buffer (150 mM NaCl, 25 mM Tris pH 7.5, 1% Triton X-100, 0.1% SDS, 0.5% deoxycholate) supplemented with protease inhibitor tablet. DNA was fragmented into 500 bp pieces using a Branson 250 sonicator. Aliquots of lysates containing 100 μg of protein were used for each immunoprecipitation reaction with the following antibodies: anti-MKL1 (Santa Cruz, sc-32909), anti-acetyl H3 (Millipore, 06-599), anti-trimethyl H3K4 (Millipore, 07-473), anti-dimethyl H3K9 (Millipore, 07-441), anti-dimethyl H3K27 (07-452), anti-TBP (Abcam, ab818), anti-TFIID (Santa Cruz, sc-273), anti-RNA Pol II (Santa Cruz, sc-899), anti-KDM7A (Biorbyt, orb67002), anti-SMAD2 (Proteintech, 12570-1), anti-SMAD3 (Abcam, ab208182), anti-SMAD4 (Proteintech, 10231-1), or preimmune IgG. Precipitated genomic DNA was amplified by real-time PCR with primers spanning the human RHOJ gene promoters. A total of 10% of the starting material is also included as the input. Data are then normalized to the input and expressed as fold changes compared with the control group.

### Scratch-Wound Healing/Migration Assay

Wound healing assay was performed as previously described (Yang et al., 2019c; Zhao et al., 2019). Cells were resuspended in serum-free media. When the cells reached confluence, scratch wound was created by using a sterile micropipette tip. Cell migration was measured 24 h after the creation of the wound and calculated by Image-Pro. Data were expressed as % migration compared with the control arbitrarily set as 100%.

### Boyden Chamber Invasion Assay

Transwell assay was performed as previously described (Yang et al., 2019b); 24-well inserts (Costar) with 10 μg/ml Matrigel (Sigma) were used for invasion assays. Cells were resuspended in serum-free media and plated into the upper chamber with the lower chamber filled with complete media. Following exposure to indicated stimuli, the cells on the upper chamber were removed. Invaded cells were stained with 0.1% crystal violet and counted. Data were expressed as % invasion compared with control arbitrarily set as 100%.

### Animals

All animal studies were reviewed and approved by the Nanjing Medical University Ethics Committee on Humane Treatment of Experimental Animals. For heterotopic xenograft, anesthetized 6- to 8-week-old SCID mice were injected subcutaneously *via* the flank with, per mouse, 5 × 10^6^ cells in phosphate-buffered saline. The mice were sacrificed 3 weeks after implantation and tumors were dissected from the mice and weighed. Tumor volume was calculated according to the following formula: 0.5 × length × width^2^. For *in vivo* metastasis, anesthetized 6- to 8-week-old SCID mice were randomly divided into different groups and injected *via* tail vein with MCF cells (1 × 10^6^ per mouse, *via* tail vein) as previously described (Wen et al., 2011; Hattori et al., 2015; Laws et al., 2020). Twenty-five days following injection, mice were sacrificed and metastasized nodules in the lungs were counted. All animal experiments were performed double-blindly.

### TCGA Data Analysis

Correlations of expression analysis and survival analysis were performed using the gepia.cancer-pku.cn web-tool and the KMplot.com web-tool, respectively. Correlations were analyzed by the Pearson correlation test. Survival rates were determined using the Kaplan–Meier method, and the significance of differences between survival rates was calculated by the log-rank test.

### Statistical Analysis

Two-sided *t*-test (for experiments involving two groups) or one-way ANOVA with *post-hoc* Scheffe analyses (for experiments involving at least three groups) was performed using an SPSS package. *p*-values smaller than 0.05 were considered statistically significant (^∗^).

## Data Availability Statement

The original contributions presented in the study are included in the article/supplementary material, further inquiries can be directed to the corresponding author/s.

## Ethics Statement

The animal study was reviewed and approved by the Nanjing Medical University Ethics Committee on Humane Treatment of Experimental Animals.

## Author Contributions

ZZ, XZ, and YY conceived the project. ZZ, BC, YZ, TZ, and YY designed and performed the experiments and collected and analyzed the data. ZZ and XZ provided the funding. YX provided the supervision and coordination. All authors contributed to the writing and editing of the manuscript.

## Conflict of Interest

The authors declare that the research was conducted in the absence of any commercial or financial relationships that could be construed as a potential conflict of interest.
