# Corrigendum: The Jumonji Domain-Containing Histone Demethylase Homolog 1D/lysine Demethylase 7A (JHDM1D/KDM7A) Is an Epigenetic Activator of RHOJ Transcription in Breast Cancer Cells

**DOI:** 10.3389/fcell.2021.729416

**Published:** 2021-07-19

**Authors:** Ziyu Zhang, Baoyu Chen, Yuwen Zhu, Tianyi Zhang, Xiaoling Zhang, Yibiao Yuan, Yong Xu

**Affiliations:** ^1^Department of Pathology, Jiangxi Maternal and Child Health Hospital, Nanchang, China; ^2^Key Laboratory of Targeted Invention of Cardiovascular Disease and Collaborative Innovation Center for Cardiovascular Translational Medicine, Department of Pathophysiology, Nanjing Medical University, Nanjing, China; ^3^School of Medicine, Nanchang University, Nanchang, China; ^4^Department of Gynecology, Jiangxi Provincial Maternal and Child Health Hospital, Nanchang, China; ^5^Institute of Biomedical Research, Liaocheng University, Liaocheng, China

**Keywords:** transcriptional regulation, epigenetics, histone demethylase, breast cancer cell, histone methylation

In the published article, there was an error in the author order, which was “Zhang Z, Chen B, Zhu Y, Zhang T, Yuan Y, Zhang X and Xu Y.” The order of the authors should have been “Zhang Z, Chen B, Zhu Y, Zhang T, Zhang X, Yuan Y and Xu Y.”

Dr. Yong Xu should not have been designated as one of the corresponding authors.

In the published article, there was an error in **Affiliations 1 and 2**. Instead of “1 Key Laboratory of Women's Reproductive Health of Jiangxi, Jiangxi Provincial Maternal and Child Health Hospital, Nanchang, China” and “2 Central Laboratory, Jiangxi Provincial Maternal and Child Health Hospital, Nanchang, China,” it should be “1 Department of Pathology, Jiangxi Maternal and Child Health Hospital, Nanchang, China.”

The authors apologize for this error and state that this does not change the scientific conclusions of the article in any way. The original article has been updated.

